# Early cryptococcosis infection in a liver transplant patient: A case report

**DOI:** 10.1002/ccr3.7699

**Published:** 2023-07-17

**Authors:** Shahrokh Sadeghi Boogar, Gholam Reza Sivandzadeh, Faezeh Sehatpour, Nazanin Dadashpour, MReza Goodarzian, Seyedeh Yasamin Parvar

**Affiliations:** ^1^ Gastroentrohepatology Research Center Shiraz University of Medical Sciences Shiraz Iran; ^2^ Gastroentrohepatology Research Center, Department of Internal Medicine, School of Medicine Shiraz University of Medical Sciences Shiraz Iran; ^3^ Department of Internal Medicine, School of Medicine Shiraz University of Medical Sciences Shiraz Iran; ^4^ Student Research Committee Shiraz University of Medical Sciences Shiraz Iran; ^5^ Molecular Dermatology Research Center Shiraz University of Medical Sciences Shiraz Iran

**Keywords:** case report, cryptococcosis, infection, liver transplantation

## Abstract

**Key Clinical Message:**

In order to early diagnose and prevent the infection dissemination in both suspected solid organ donors and recipients after transplantation, pretransplantation screening tests for rare etiologies like *Cryptococcus neoformans* should be necessitated, as they can affect many vital organs, especially the brain, liver, and lungs.

**Abstract:**

Cryptococcosis is a systemic fungal infection mainly affecting immunocompromised patients. The infection is occasionally seen in 16–21 months after organ transplantation, while early involvement is uncommon within <30 days posttransplantation. In the present study, we reported an unusual case of cryptococcosis infection 21 days after transplantation, limited to the transplanted liver in a 60‐year‐old male. Treatment with an antifungal agent showed prompt improvement in his clinical condition.

## 1 INTRODUCTION

Cryptococcosis is a systemic fungal infection caused by an opportunistic pathogen that primarily affects immunocompromised individuals. The main cause of infection in the host is the encapsulated yeast *Cryptococcus neoformans* (Division Basidiomycota), which has four serotypes. Fungus concentrations are high in nitrogen‐rich air, and the primary mode of transmission is inhalation; however, it can also spread through the skin and solid organs upon transplantation in rare circumstances.^1^, ^2^

According to recent data, the mortality rate of cryptococcosis infection is significantly higher in immunocompromised individuals, ranging from 20% to 100% in organ‐transplanted patients.^3^, ^4^ The disease's clinical manifestations range from asymptomatic colonization in the respiratory airway to pulmonary disease, meningitis, skin disease, and eye disease. Even though the clinical presentation is restricted to a single anatomical location, the absence of pulmonary or non‐meningeal infection does not exclude widespread infection.^5^

Cryptococcosis is one of the most invasive mycoses in solid‐organ transplant (SOT) recipients. Infection typically appears 16–21 months after transplantation. Moreover, the majority of the SOT recipients with cryptococcosis exhibited serological evidence of infection before transplantation.^6^, ^7^ The most common sites of cryptococcosis in SOT recipients are the central nervous system (CNS), lungs, and skin. In a recent study, up to 5% of SOT recipients with cryptococcosis had a very early infection within less than 30 days posttransplantation, which generally indicates preexisting cryptococcus infection. While two vital organs including the liver and lung are the most common sites for this early infection, involving the former one is with much worse complications. Furthermore, the very early disease is generally found in an unusual site, such as the transplanted allograft or the surgical fossa. Transplanted organ involvement usually suggests transmission from the donor, and it is most common in liver transplants. This finding may be related to the susceptibility of liver transplant recipients to cryptococcosis during transplant candidacy.^8^, ^9^

In the present study, we reported a rare and unusual case of a liver transplant in our centers, who presented with liver mass in ultrasonography in his routine follow‐up after transplantation. The liver mass biopsy revealed early cryptococcosis infection.

## 2 CASE REPORT

A 60‐year‐old man, known case of liver cirrhosis due to hepatitis B with orthotopic liver transplantation—from a cadaveric 55‐year‐old donor with normal routinely checked pretransplant laboratory data who died of a brain stroke—was presented 40 days after transplantation. Tacrolimus (PROGRAF®, Hartkapseln, 1 mg capsule, twice daily), Myfortic (Mycophenolsäure, 360 mg tablet, twice daily), tenofovir (Virbit® 300 mg tablet, daily), and folic acid (Mehrdarou 5 mg tablet, daily) were all being administered to the patient at home. A 20*15 mm well‐defined hyper‐echogenic lesion was found in the inferior capsule of the right transplanted liver lobe during posttransplant ultrasonography follow‐up (21 days later). In order to rule out other possible diagnoses, including autoimmune hepatitis and viral hepatitis, a true cut biopsy was performed under the guidance of a computed tomography (CT). This revealed extensive necrosis containing many fungal elements in favor of cryptococcosis. Cryptococcosis was also detected using the Grocott Methenamine Silver stain (GMS).

His vital sign was steady upon admission to the hospital and no signs of fever were detected. The other part of the physical examination did not reveal any abnormal findings. Detailed laboratory results are shown in Table 1.

**TABLE 1:** Laboratory data of a 60‐year‐old male presented with cryptococcosis infection 24 days after a liver transplantation.

Lab data	Date				
	2022.01.07	2022.01.08	2022.01.16	2022.01.26	2022.02.09
WBC (×1000/mm^2^)	4.6	4	‐	5.6	‐
Hemoglobin (g/dL)	13.9	13.1	‐	12.8	‐
MCV (fL)	83.9	83	‐	82.9	‐
Platelet (×1000/mm^2^)	196,000	212,000	‐	250,000	‐
BUN (mg/dL)	17	20	26	23	20
Creatinine (mg/dL)	1.4	1.4	2.6	2.3	2
Sodium Na (meq/L)	142	142	139		139
Potassium K (meq/L)	4.7	4.4	‐	4.1	3.9
PT	13.7	‐	‐	‐	‐
PTT	32	‐	‐	‐	‐
INR	1.05	‐	‐	‐	‐
AST (U/L)	24	‐	‐	19	‐
ALT (U/L)	14	‐	‐	13	‐
ALK.P (U/L)	243	‐	‐	271	‐
Albumin (g/L)	4.2	‐	‐	3.9	‐
Total bilirubin (mg/dL)	0.6	‐	‐	0.67	‐
Direct bilirubin (mg/dL)	0.1	‐	‐	0.17	‐

Abbreviations: ALK.P, alkaline phosphatase; ALT, alanine amino transferase; AST, aspartate amino transferase; BUN, blood urea nitrogen; CMV, cytomegalovirus; INR, international normalized ratio; MCV, mean corpuscular volume; PT, prothrombin time; PTT, partial thromboplastin time; PCR, polymerase chain reaction; WBC, white blood cell.

Moreover, cytomegalovirus (CMV) and COVID‐19 (Corona virus disease‐2019) polymerase chain reaction (PCR) were negative. After the consultation with an infectious specialist, liposomal amphotericin B (ExirNano Sina 5 mg/kg/day IV) was started. Moreover, the serum sample for cryptococcus antigen and antibody was taken from the patient and surprisingly were positive, which could imply acute cryptococcosis in the recipient.

It is noteworthy that on a review of the recipient's past data, the patient did not have any clinical evidence of any infection. Serum creatinine increased from 1.4 to 2.6 mg/dL during the course of admission. After a week of treatment, amphotericin was switched to fluconazole (AVESINA 800 mg tablet, daily for 4 weeks). Additionally, while in the hospital, he complained of a new onset pulsatile non‐migrating headache in the frontal and orbital areas; therefore, the possibility of immune reconstitution inflammatory syndrome (IRIS) and CNS infections was considered. Both a brain CT scan and a paranasal sinus (PNS) CT scan were carried out. The result of the brain CT was completely normal whereas, the PNS CT scan revealed a lesion in the right maxillary sinus. The results of the maxillary sinus and nasal cavity biopsies performed at the ear–nose–throat (ENT) visit were normal. Additionally, the lung CT scan also did not reveal any abnormal findings. After 35 days of treatment initiation, the abdomen‐pelvic magnetic resonance imaging (MRI) was completely normal and showed no sign of liver occupying lesion.

Finally, he was discharged in fair condition with oral fluconazole and his other previous medications.

## 3 DISCUSSION

Cryptococcosis is an uncommon infection in the general population that causes serious complications, especially neurologic ones in immune‐compromised patients including human immunodeficiency virus (HIV), malignancy, organ transplant, and in cirrhotic patients. Early infection within 30 days posttransplantation is unusual, although this infection occasionally occurs in 16–21 months following organ transplantation.^9^, ^10^ According to the Chang et al. report, a 63‐year‐old lady who had hyperbilirubinemia during her check‐up about 2 weeks after transplantation had cryptococcosis found in her liver biopsy. *Cryptococcus neoformans* was detected in her blood culture as well.^11^

We presented a case with liver involvement of cryptococcosis 24 days after a liver transplant. Based on the previous studies, blood group and rhesus (Rh) type, basic metabolic evaluation, complete blood count (CBC) and differential, liver enzyme and coagulating parameters, serum calcium and vitamin D level, urinalysis, urine drug screening, hepatitis A, B, and C serology, HIV, varicella zoster virus (VZV), CMV, Epstein–Barr virus (EBV), rapid plasma reagin (RPR), mumps, measles, rubella, and latent tuberculosis screening were done as routine laboratory tests before liver transplantation. Furthermore, our centers do not routinely assess the pretransplant screening for cryptococcosis.^12^

The diagnosis of cryptococcosis in our cases was based on liver lesion biopsy which was incidentally found during posttransplant hepatobiliary ultrasonography. The infection was limited to the transplanted organ and no other organs (lung, skin, brain, etc.) were involved. Besides that, our case's immune‐deficiency state was a potential risk factor for early cryptococcal infection. Finally, treatment with an antifungal agent showed prompt improvement in his condition.

According to the report conducted in Brazil in 2020, disseminated cryptococcosis infection was detected in a patient presenting with fever, headache hyponatremia, and elevated aminotransferases enzyme level. Additionally, multiple brain lesions were detected in favor of cryptococcosis in the patient's brain MRI. Disseminated cryptococcosis was also detected in two cases who received a transplanted kidney from the mentioned patient as a donor.^13^ It is in contrast with our study as the constitutional symptoms were more prominent in two previous cases rather than in our presented patient.

After candidiasis and aspergillosis, cryptococcosis is the third most common cause of fungal infection among organ‐transplanted patients. The primary mechanism of infection in many individuals, particularly in populations with late infection (more than a year after transplant), is the reactivation of latent infection in recipients. Early infection, however, is primarily brought on by a contaminated donor.^11^, ^14^, ^15^

It is noteworthy that undiagnosed diseases can be found in donor‐derived solid organs. We consider donor‐derived cryptococcosis to be a differential diagnosis for recipient cryptococcosis, which may result in the same condition in the recipient patient. As a result, it is even more critical to include a cryptococcosis serology test as a routine pretransplant examination for solid organ donors.

## 4 CONCLUSION

Early cryptococcosis is one of the important fungal infections in SOT patients. It should be considered when the recipients have symptoms of infection (malaise, fever, high erythrocyte sedimentation rate [ESR], and C‐reactive protein [CRP]), abnormally high liver function test (LFT) index, pain and mass palpation in right upper quadrant (RUQ) within 30 days after SOL transplantation. Further studies with large samples are needed to evaluate the pathophysiology, etiology, risk factors, and outcome of early posttransplant infection.

## AUTHOR CONTRIBUTIONS

**Shahrokh Sadeghi Boogar:** Conceptualization; data curation; methodology; supervision. **Gholam Reza Sivandzadeh:** Conceptualization; data curation; methodology; project administration; supervision. **Faezeh Sehatpour:** Visualization; writing – original draft. **Nazanin Dadashpour:** Visualization; writing – original draft. **MReza Goodarzian:** Validation; writing – review and editing. **Seyedeh Yasamin Parvar:** Writing – original draft; writing – review and editing.

## FUNDING INFORMATION

None.

## CONFLICT OF INTEREST STATEMENT

The authors certify that there is no conflict of interest with any financial organization regarding the material discussed in the manuscript.

## ETHIC STATEMENT

The manuscript has been approved by all authors and has never been published or under the consideration for publication elsewhere. We confirm that tables are original and created by authors. We guarantee that all authors listed on the title page have read the manuscript and attest to the validity and legitimacy of the data. We would also like to undertake that we have read the plagiarism policy and submitting the article with complete responsibility. This study was done in compliance with the Declaration of Helsinki and approved by the ethics committee of the university. The patient gave his informed consent prior to his inclusion in the study.

## CONSENT

In compliance with the journal's patient consent policy, written informed consent was obtained from the patient in order to publish this report.

## TRANSPARENCY STATEMENT

This manuscript is an honest, accurate, and transparent account of the study being reported; no important aspects of the study have been omitted; and any discrepancies from the study as registered have been explained.

## Data Availability

The data supporting the findings of this study are available on request from the corresponding author. The data are not publicly available due to privacy or ethical restrictions.
